# Elucidating the structural dynamics induced by active site mutations in 3C protease of foot-and-mouth disease virus

**DOI:** 10.1371/journal.pone.0321079

**Published:** 2025-04-21

**Authors:** Sthitaprajna Sahoo, Hak-Kyo Lee, Donghyun Shin

**Affiliations:** 1 Department of Agricultural Convergence Technology, Jeonbuk National University, Jeonju, Republic of Korea; 2 Department of Animal Biotechnology, Jeonbuk National University, Jeonju, Republic of Korea; Chung-Ang University, KOREA, REPUBLIC OF

## Abstract

The viral replication of foot-and-mouth disease virus (FMDV) and other picornaviruses primarily depends on the successful processing of a polyprotein precursor by the enzyme 3C protease (3Cpro) at specific sites. The crucial role of 3Cpro in viral replication and pathogenesis makes it a potential target for developing novel therapeutics against foot-and-mouth disease. The β-ribbon region (residues 138–150) containing the active site residues (C142) in 3Cpro is found to be conserved and contributes significantly to substrate specificity. Moreover, experimental reports suggest that mutations at position 142, particularly C142S and C142L, exhibit different functional activities. However, the intrinsic dynamics and conformational changes induced by active-site mutations of 3Cpro remain unclear, limiting the development of novel inhibitors of 3C protease. Accordingly, we carried out molecular dynamics (MD) simulations with multiple replicates for both the WT and mutants of 3Cpro. The observed results suggest that the C142S mutant induces substantial structural transitions compared to the WT and C142L. In contrast, the essential dynamics of the mutants significantly varied from those of the WT 3Cpro. Moreover, cross-correlation analysis revealed a similar pattern of anti-correlation between the amino acid residues of the WT and C142L mutant complexes. Analysis of the betweenness centrality of the WT and the mutants from the residue interaction networks revealed common residues for intra-residual signal propagation. The results from our study suggest that the active site mutant C142S may induce conformational changes, which can cause the β-ribbon region to bend towards the catalytic pocket and inhibit the enzymatic activity. C142L substitution may also alter the β-ribbon region conformation, which may impact the substrate binding process during proteolysis, as reported in previous studies. These results can provide a better understanding of the conformational dynamic behavior of 3Cpro active-site mutants and may assist in developing potential inhibitors against foot-and-mouth disease.

## 1. Introduction

Diseases associated with RNA viruses are often challenging to control because of their high mutation rates and the constant emergence of new variants. Genetic and antigenic variations among viruses determine the extent to which the immunity developed against one variant will be effective against another. Foot-and-mouth disease virus (FMDV) is an antigenically variable, highly infectious virus that infects a wide variety of cloven-hoofed animals, including cattle, goats, sheep, and pigs [1,2]. Over the years, Foot-and-mouth disease (FMD) outbreaks have occurred worldwide and have significantly affected the global livestock industries [3]. This disease is caused by a single-stranded positive-sense RNA virus belonging to the Picornaviridae family, with seven serotypes: O, A, C, Asia1, and SAT1–3. The O serotype is prominent in most FMD outbreak regions, including South America, Africa, Asia, the Middle East, and India [4]. Treatment of FMD mainly focuses on vaccination (using inactivated virus particles) against the prevailing strain of FMDV. Although vaccination against FMDV may slow the spread of some FMD outbreaks, it requires several days to induce an immune response and does not completely stop the virus from spreading [5,6]. As no particular treatment is available and it is challenging to match the virus vaccine to different serotypes, exploring alternative methods, such as antiviral treatment is essential. Research and development of such therapies require a thorough understanding of the molecular mechanisms underlying viral replication and the structural details of the target protein.

The enzyme 3C protease (3Cpro) is a highly conserved protein in FMDV and is mainly composed of 207 amino acids. It is responsible for 10 of the 13 cleavages by targeting particular sequences within a polyprotein [7–9]. After entering host cells, the viral genome is translated into a long polyprotein of 2,300 amino acids. The virus-encoded protease cleaves this polyprotein precursor to produce 14 distinct proteins that are essential for RNA replication and production of new viral particles [10]. Additionally, 3Cpro cleaves host proteins, such as eIF4G, that are present in infected cells and suppresses cellular transcription by cleaving histone H3 protein [11,12]. The vital role of the 3C protease in viral replication and cleavage activity makes it a potential drug target for the development of antiviral drugs against FMDV.

**Fig 1:**
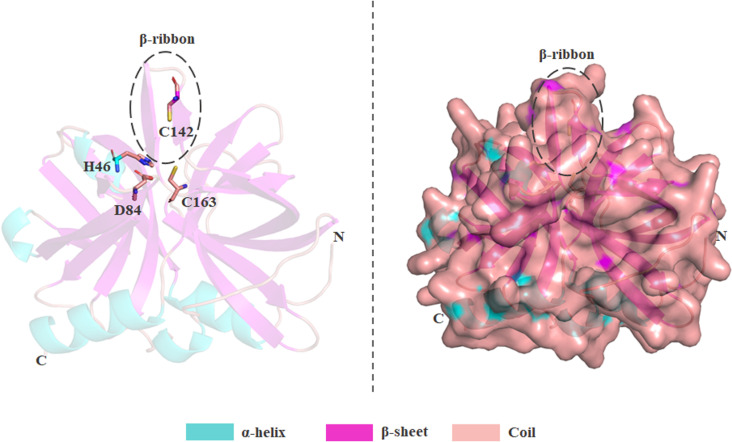
Three-dimensional structure of the 3Cpro enzyme of foot-and-mouth disease virus (FMDV). Catalytic triad residues (H46, D84, and C163) and active site residue (C142) are highlighted in the stick model, and the conserved β-ribbon structure is shown in a dashed circle. The alpha-helixes, β-sheets, and loops of the protein are represented with cyan, magenta, and salmon color, respectively.

Several structural studies reported an overall fold in picornaviral 3Cpro, which is very similar to the structure of chymotrypsin-like serine proteases [13–15]. Unlike other serine proteases, FMDV 3Cpro acquires a Cys-His-Asp/Glu catalytic triad in its active site instead of a Ser-His-Asp triad [16]. Previous studies have shown that the third amino acid of the catalytic triad plays a significant role in the picornaviral 3Cpro. This is consistent with the strict conservation of the residue as aspartic acid (D) or glutamic acid (E) in 3Cpro sequences and also suggests that substituting this residue has a severe adverse impact on catalytic activity [17,18]. In the initial structure proposed for FMDV 3Cpro, the β-ribbon region (residues 138–150) was described as disordered, and this is where the active site is located (Fig 1). However, this conserved β-ribbon was observed to fold over the peptide-binding cleft in other picornaviruses 3Cpro [19–21]. This phenomenon was further studied, and a refined FMDV 3Cpro crystal structure was solved with an amino acid substitution (C142S) to make the protein soluble; the results suggest that this β-ribbon region is also conserved in 3Cpro of FMDV [14,22]. Despite having some dynamic nature, the β-ribbon region directly interacts with the peptide in the active site and contributes significantly to substrate specificity [23]. Despite all these previous studies, a key knowledge gap remains regarding the extent to which active-site mutations, such as C142S and C142L, affect the conformational dynamics and allosteric regulation of 3Cpro. As it has been demonstrated that mutagenesis of C142 at the apical-tip of the β-ribbon structures significantly affects the catalytic activity, this loop most likely serves to orient the substrate for proteolysis. Although structural studies have recognized this β-ribbon flexibility, the impact of specific mutations in this region on the enzyme’s overall stability, substrate interactions, and function properties remains largely unexplored. Hence, to address this gap, studies on the intrinsic dynamics induced by these active site mutations, such as C142S and C142L, can provide detailed structural insights at the molecular level, which may be helpful in developing antiviral treatments against FMD by targeting the 3Cpro protein. In this study, we assessed wild-type 3Cpro (WT) and its active-site mutants (C142S and C142L) through 200 ns molecular dynamic (MD) simulations with three independent replicates (total of 600 ns each). Furthermore, we performed essential dynamics or principal component analysis (PCA) and residual network analysis (RIN) to better understand the collective motion and intra-residual signaling induced by these single-point mutations. By integrating these computational approaches, we provide a comprehensive characterization of how C142S and C142L mutations modulate the structural integrity and functional impact of 3Cpro.

## 2. Materials and methods

### 2.1. Structural preparation of WT and mutants of 3Cpro

The crystal structures of FMDV 3Cpro available in the RCSB Protein Data Bank (http://www.rcsb.org) contain mutations at their active site residues (PDB ID: 2J92, 2WV4, 2BGH). As no crystal structure is available for wild-type FMDV 3Cpro, we retrieved the FASTA sequence from the UniProt database (https://www.uniprot.org/) with UniProt ID P03306 (residues: 1650–1856) and modeled the WT 3Cpro structure using AlphaFold2, the most advanced deep learning model for predicting protein folding [24]. In addition, we downloaded the C142S and C142L mutant complexes with PDB IDs 2J92 and 2WV4, respectively, and used them as templates to predict the structure of the WT complex [15,22]. The standard AlphaFold2 pipeline was employed using ColabFold to predict the 3Cpro structure. The pLDDT scores for most residues ranged from 80 to 100, indicating a high-confidence backbone prediction. However, as expected, lower confidence scores were observed at the N-terminal and C-terminal regions, which is common due to their inherent flexibility. Furthermore, to construct the mutant models, we used the WT model and altered cysteine to serine (C142S) and cysteine to leucine (C142L) at residue 142 with probable rotamers using the Mutagenesis Wizard in PyMOL. Lastly, we validated the model structures via their stereochemical properties and Ramachandran plot (S1 Fig) using the SAVESv6.0 webserver (https://saves.mbi.ucla.edu/) [25–27]. Moreover, we assessed the evolutionary conservation of each amino acid of 3Cpro using the ConSurf web server. Furthermore, we assessed the evolutionary conservation of each amino acid in 3Cpro using the ConSurf web server. The analysis revealed that all catalytic residues, H46, D84, and C163, are highly conserved throughout evolution (S2 Fig). However, due to limited available data on the active site residue C142, the server was unable to provide sufficient information for its conservation status.

### 2.2. Molecular dynamic (MD) simulations

All the prepared structures (WT, C142S, and C142L) were subjected to all-atom MD simulations using GROMACS 2022.2 with the AMBER-ff99SBILDN forcefield [28,29]. The topology files were prepared using the *gmx pdb2gmx* module and placed in a dodecahedral box with explicit water molecules. A total of 8604, 8318, and 8255 TIP3P water molecules were added to the WT, C142S, and C142L, respectively, with a periodic boundary of 1.0 nm. Three chlorine (Cl^-^) ions in each were added to neutralize all systems using the *gmx genion* module. A maximum tolerance of 1,000 kJ/mol/nm was set for energy minimization at every 100 ps using the steepest descent optimization algorithm, as mentioned in our previous studies [30,31]. Subsequently, NVT and NPT equilibrium simulations were performed for 100 and 500 ps, respectively. A temperature of 300K and pressure of 1 bar were maintained using a Berendsen thermostat (V-rescale) and the Parrinello-Rahman pressure coupling algorithm, respectively [32,33]. The NVT phase stabilizes the system at the desired temperature using a thermostat. It allows atoms to adapt to the simulation conditions without fluctuations in volume, ensuring proper kinetic energy distribution. Next, the system was subjected to constant pressure through NPT to adjust the density and volume to realistic conditions. This step ensures that the system reaches equilibrium density before the production phase. A cut-off of 1.0 nm was set to maintain short and long-range electrostatic interactions, and the Particle Mesh Ewald (PME) algorithm was used to sustain long-range interactions [34]. Furthermore, all bonds were restrained using the LINCS algorithm [35,36]. Lastly, three independent replicate simulations of 200 ns each were performed using different random velocities for all systems, where 2 fs time steps were used and 50 ps coordinates were saved for the entire trajectory. Most analyses were performed using built-in GROMCAS modules with concatenated trajectories from replicate simulations.

### 2.3. Principal components analysis (PCA)

The global concerted motions that occurred during MD simulations were assessed using essential dynamics (EDs) or principal component analysis (PCA) [37,38]. The principal components (PCs) of all systems with dominant motions were calculated on the concatenated trajectories using 50 ps coordinates from the last 100 ns simulations of three replicate trajectories. We also removed rotational and translational movements and constructed a covariance matrix using the *gmx covar* module of gromacs, as reported in our previous studies [31,39,40]. The essential subspace can be defined as a subset of PCs that distinguish between conformations based on their distinct structural similarities and dissimilarities. Hence, the first 10 PCs were considered to define the essential subspace by calculating the root mean square inner product (RMSIP) and comparing the WT with the mutant systems [41,42]. The RMSIP is the cumulative overlap among all pairs of *l* largest eigenvectors and can be calculated as:


$$RMSIP={\left(\frac{1}{l}\overset{l}{\underset{i=1}{\sum }}\overset{l}{\underset{j=1}{\sum }}{\left(n_{i}^{A}.n_{j}^{B}\right)}^{2}\right)}^{\frac{1}{2}}$$

where $n_{i}^{A}$ and $n_{j}^{B}$ denote the *i*^th^ and *j*^th^ eigenvectors from systems A and B, respectively, and *l* denotes the dimensionality of the subspace (*l*=10 in this case). The RMSIP values ranged from 0 to 1, representing the orthogonal and identical directionality of the essential subspace, respectively. In addition, we have evaluated the Gibbs free energy landscape (FEL) with the first two PCs (PC1 and PC2) using the *gmx sham* module of gromacs, and plots were generated using Mathematica version 12. Furthermore, we generated a dynamic cross-correlation map (DCCM) between the protein residues using Bio3D and analyzed the residual correlations (correlated or anticorrelated) in all three systems using PyMOL.

### 2.4. Residue interaction networks (RIN)

Substitution of an amino acid in a protein structure can significantly affect the network of interactions, stability, and protein folding. The impact of single or multiple mutations on protein structure can be studied through a detailed RIN. Hence, we used the NAPS webserver (http://bioinf.iiit.ac.in/NAPS/) to generate RINs for both the WT and mutants, where residues and contacts were represented by nodes and edges, respectively [43,44]. The threshold limit was set for 0–7Å using Cα pair residual network type and unweighted edge. We calculated the betweenness centrality value (C_B_ value), which indicates residues that are important for the protein function and allows the prediction of the central residue (node) for signal transduction.

## 3. Results and discussion

### 3.1. Structural dynamics and stability of WT and mutants of 3Cpro

**Fig 2:**
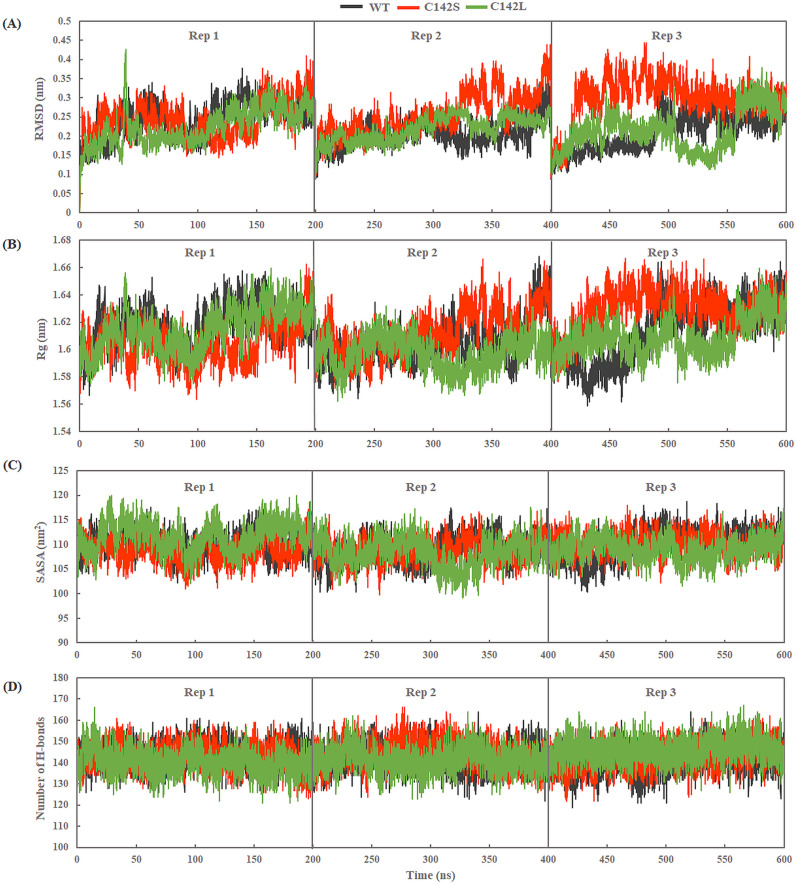
Structural stability analysis of WT and mutants of 3Cpro. (A) RMSD, (B) Rg of backbone atoms. (C) SASA and (D) Number of Intra H-bonds throughout the simulation. Black, red, and green represent WT, C142S, and C142L models of 3Cpro.

**Fig 3:**
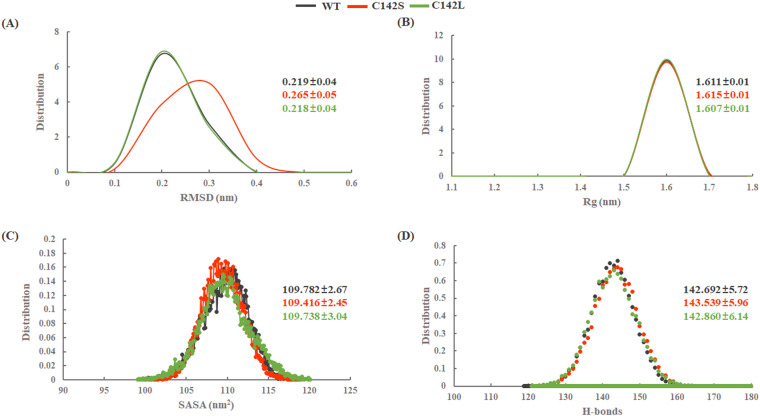
Probability density distributions from the 600 ns concatenated trajectories. (A) Root means square deviation (RMSD); (B) Radius of gyration (Rg); (C) Solvent-accessible surface area (SASA); and (D) intra H-bonds. Average and standard deviation values are represented according to their respective colors. Black, red, and green represent WT, C142S, and C142L models of 3Cpro.

All three systems (WT, C142S, and C142L) were prepared and subjected to three independent replicate simulations of 200 ns each (total of 600 ns) at different initial velocities. To assess the structural and conformational stability of all three complexes, the root mean square deviation (RMSD) values of the protein backbone were calculated with reference to their respective initial structures. RMSD analysis revealed that all systems stabilized after ~50 ns of MD simulation. Replicates 1 and 2 (Rep1 and Rep2) of both WT and C142L showed RMSD fluctuations similar to those of Rep3 (Fig 2A). However, in Rep3, WT and C142S showed lower deviation towards the last 100 ns of simulation, with an RMSD range of 0.2–0.35 nm, whereas C142L fluctuated slightly, with an RMSD range of 0.15 to 0.35 nm in the last 100 ns of simulation. In addition, we calculated the probability density distribution of the RMSD values from the concatenated trajectories (total 600 ns) and represented in (Fig 3A). The unimodal distribution from the cumulative trajectories yielded average RMSD values of 0.219±0.04, 0.265±0.05, and 0.218±0.04 nm for WT, C142S, and C142L, respectively, which indicates a distinct RMSD distribution for C142S as compared to WT and C142L.

The radius of gyration (Rg) of the backbone atoms for both the WT and mutants was calculated to predict the spatial distribution of the protein and to assess compactness or structural stability throughout the MD simulation. The Rg values of all systems were calculated and are presented in (Fig 2B). The C142S mutant complex showed a relatively higher Rg value, as compared to the WT and C142L complexes. Moreover, the C142L mutation exhibited lower Rg values, indicating higher stability than other mutations. The probability density distribution of Rg clustered at 1.611±0.01, 1.615±0.01, and 1.607±0.01nm in the case of the WT, the C142S, and C142L mutations, respectively (Fig 3B). However, the differences in Rg values indicate that in comparison with C142L, the C142S mutation in 3Cpro may exhibit noticeable conformational changes. Furthermore, we calculated the solvent-accessible surface area (SASA) for all three systems to analyze the solvent-exposed area of the protein throughout the MD simulation (Fig 2C). The SASA values for the WT and mutants were in the range of 105–115 nm^2^. The probability density distribution of SASA values exhibited an average spatial distribution of 109.782±2.67, 109.416±2.45, and 109.738±3.04 nm^2^ for WT, C142S, and C142L mutations, respectively (Fig 3C). Based on the observed SASA values, the C142S and C142L mutations did not cause any significant changes in the solvent accessibility of the protein compared with the WT.

Hydrogen bonds play an important role in maintaining the structural integrity and stability of proteins [45]. The RMSD and Rg values of the WT and mutants differed slightly in each of the three replicates, which may be due to the formation and breakage of intramolecular H bonds. Hence, to address this issue, we calculated the intra H-bonds throughout the simulation using the *gmx hbond* tool in GROMACS (Fig 2D). The intra H-bonds analysis revealed the probability density distribution of H-bonds clustered at a value of 142.692±5.72, 143.539±5.96, and 142.860±6.14 for WT, C142S, and C142L respectively (Fig 3D). No significant changes were observed in the number of intra H-bonds for either the WT or mutants. However, a slightly higher number of intra H-bonds was observed for the C142S mutant system, which indicates that the substitution of cysteine with serine induces structural changes that may inhibit the catalytic activity of the protein.

The MD simulations provided valuable insights into the structural stability of the WT, C142S, and C142L variants of 3Cpro. RMSD analysis demonstrated that all three systems stabilized after ~50 ns, with WT and C142L exhibiting similar fluctuations, while C142S exhibited a distinct RMSD distribution, suggesting differences in its conformational behaviour. The Rg analysis further supported these observations, revealing that the C142L mutant maintained a more compact structure, indicating higher structural stability, whereas C142S displayed slightly increased Rg values suggesting enhanced flexibility. This finding is consistent with previous studies on picornaviral 3Cpro, which have reported that mutations in key structural regions can alter protein stability and enzymatic function [16]. The β-ribbons region where these mutations are located, has been recognized for its role in substrate binding and catalytic activity [14]. Our results reinforce the idea that this region is structurally and functionally critical. Moreover, the comparative SASA values among these variants indicate that while these mutations impact internal conformational dynamics, they do not significantly alter solvent exposure. Overall, these findings suggest that C142L enhances structural stability, whereas C142S induces greater conformational variability, potentially impacting enzymatic activity. These results align with previous structural studies on viral cysteine proteases, which indicate that mutations at key catalytic and structural sites can significantly affect proteolytic function.

### 3.2. Residual flexibility and change in secondary structure analysis

**Fig 4:**
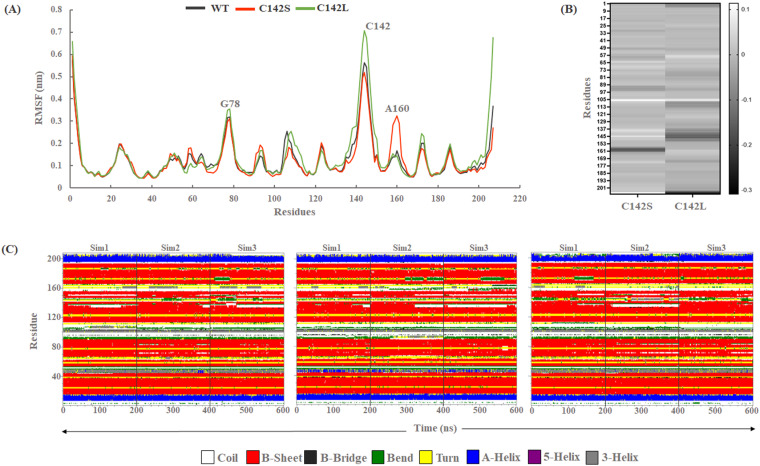
Residual flexibility throughout the MD simulation. (A) RMSF of backbone atoms from the concatenated trajectory. (B) Heatmap representing △RMSF of C142S and C142L relative to WT. (C) DSSP plot represents the changes in secondary structure for WT, C142S, and C142L systems.

The fluctuations in each amino acid residue during the entire simulation were assessed using root mean square fluctuation (RMSF) analysis. The RMSF values were calculated for the backbone atoms from the concatenated trajectory, as shown in (Fig 4A). RMSF analysis revealed that except for the N and C terminal regions, residues 76–80, conserved β-ribbon region containing active site residue, and residues 156–161 exhibited high residual flexibility, as compared to other residues. However, no residual fluctuations were observed in the catalytic residues (H46, D84, and C163) of any of the three systems. The β-ribbons region having active site residues exhibited higher fluctuations in C142L compared to the WT and C142S mutant systems. The average residual fluctuations for WT, C142S, and C142L were calculated to be 0.126, 0.124, and 0.144 nm respectively, indicating a larger residual movement in the C142L mutant model compared to the other systems. In addition, we have calculated the △RMSF value for the C142S and C142L mutants relative to WT. △RMSF is defined as the difference between the RMSF of wild-type protein and the RMSF of the mutants. A positive value of △RMSF indicates a rigid zone while negative △RMSF values indicate a flexible zone with a dark color (Fig 4B). The dark grey color in the △RMSF heatmap for C142L relative to WT clearly indicates the flexibility of the conserved β-ribbon linker which was reasonable because the substitution of cysteine (smaller amino acid) to leucine (bigger amino acid) at position 142 may cause conformational changes in the active site pocket. Additionally, C142S did not exhibit any active site residual fluctuations but showed an atypical fluctuation at A160.

The secondary structure of a protein plays a crucial role in determining its overall structure and, consequently, its function. Understanding the role of secondary structures is crucial for gaining insight into the functions of proteins. Researchers often use various experimental and computational techniques to study and predict protein secondary structures [46]. Alpha helices (α) and beta sheets (β) are examples of secondary structural elements that are typically stable, whereas coils, turns, and loops are flexible. In the current study, the Define Secondary Structure of Proteins (DSSP) was used to evaluate and understand the changes in the secondary structure of the WT and mutants throughout the simulation (Fig 4C). The overall changes in the β-sheets throughout the simulation were calculated to be 50.76%, 51.04%, and 51.17% for WT, C142S, and C142L respectively. Similarly, 8.11%, 8.80%, and 7.86% of changes in the α-helices were observed for WT, C142S, and C142L respectively (S1 Table). Secondary structure analysis showed that the stable conformation was preserved for both the WT and mutants of 3Cpro during the simulation period.

The observed differences in flexibility and structural stability among these variants highlight the critical role of the β-ribbon region in maintaining the functional integrity of 3Cpro. The increased flexibility in the β-ribbon for specific mutations, particularly C142L, emphasizes the potential impact of residue substitutions on the active site’s conformational dynamics and substrate interactions. Despite these changes, the overall secondary structure remained stable across all three systems, suggesting that 3Cpro can tolerate certain mutations without losing its global structural integrity. These results advance our knowledge of how active site mutations influence the dynamics and functionality of viral proteases, providing insights that could guide future antiviral drug design targeting FMDV 3Cpro.

### 3.3. Essential dynamics of WT and mutants of 3Cpro

PCA is a dimensionality reduction technique widely used in various fields, including MD simulations of protein complexes. PCA can provide crucial insights into the structural dynamics and functional implications of the wild-type (WT) and mutant (MT) protein complexes. Identifying the principal components (PCs) associated with large eigenvalues, which correspond to the most dominant motions, can provide structural insights into the functional dynamics that may be altered by mutations. In addition, it can assist in identifying key residues or regions responsible for observed conformational changes [37,47,48]. Hence, to assess the collective motions that contribute significantly to the conformational changes induced by these mutations, PCA was performed using concatenated trajectories (last 100 ns trajectories from each replicate; total 300 ns).

**Fig 5:**
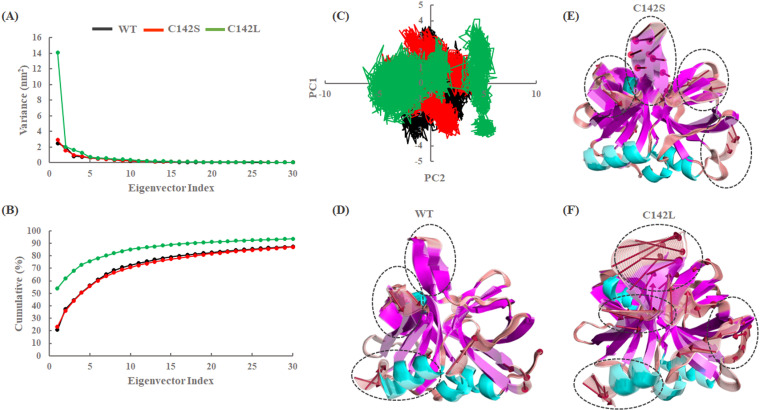
Collective mode analysis from the concatenated trajectories. (A) Eigenvalues of all the systems. (B) Cumulative percentage from the first 30 eigenvectors. (C) Projection of first two PCs. Black, red, and green colors represent the WT, C142S, and C142L systems of 3Cpro. (D, E, F) Structural changes in WT, C142S, and C142L with respect to PC1 are represented using extreme conformations with 30 frames. Black-dashed circles represent large conformational changes, and red arrows represent the size and direction of movements.

The results from the PCA revealed diagonalized co-variances of 12.05, 12.52, and 26.15 for the WT, C142S, and C142L systems, respectively, which indicates a higher fluctuation of the C142L system, compared to the others. Moreover, the first three eigenvectors had the most significant motions, with cumulative percentages of 44.42%, 43.90%, and 68.01% for the WT, C142S, and C142L, respectively (Fig 5A and 5B). A 2D projection was generated by assessing the first two PCs (Fig 5C), which showed that both the WT and C142S exhibited a similar type of spread with a low energy barrier, whereas C142L exhibited a large spread projection in the phase space. Furthermore, similarities and dissimilarities between the essential subspaces of the WT and mutants were defined using the first 10 PCs, which contributed significantly to more than 70% of the overall covariance. The RMSIP calculations highlighted the essential subspace of the mutant ensembles in comparison with the WT. The RMSIP calculation between the WT and C142S mutant of 3Cpro exhibited a normalized overlap of 0.554, whereas, for WT vs. C142L, it was calculated to be 0.382. S3A Fig represents the pairwise comparison plot between the WT and 3Cpro mutants, suggesting that the mutants exhibited distinct collective motion compared to the WT with no substantial overlap. In particular, the RMSIP of the first three eigenvectors, which comprise the majority of the dominant motions, differ significantly. Hence, the distribution of all systems was projected using the first three PCs and shown in S3B Fig. In addition, we assessed variations in the global motion of the WT and mutants by considering the extreme conformations with 30 frames from the first PC of the concatenated trajectories (Fig 5D,5E, and 5F). The results revealed that, as compared to the WT, both the mutants showed larger fluctuation in the β-ribbon linker region. In the case of the C142S mutant system, a larger fluctuation was observed in the active site residue (S142) and catalytic residues (H46, D84, C163), which causes the β-ribbon structure to bend towards the catalytic pocket and close the substrate binding site. Although no significant changes were observed in the catalytic residues of the C142L mutant system, the β-ribbon structure was observed to be bent away from the catalytic pocket, which might be the reason for reducing enzymatic activity.

**Fig 6:**
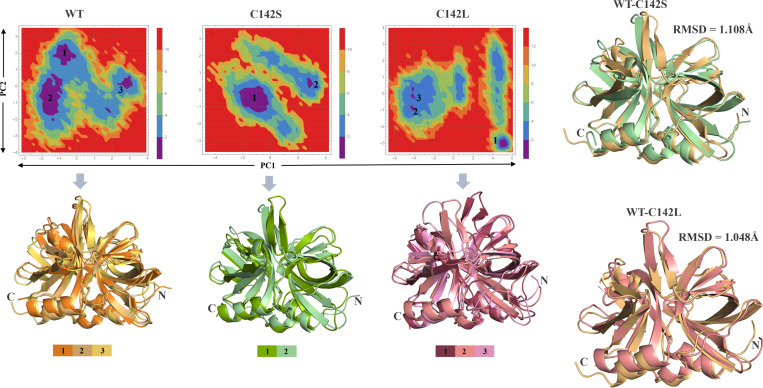
Free energy landscape of all three systems and their representative structures. The low-energy structures were retrieved from their respective global minima and superimposed. In addition, the superimposition of WT and representative mutant conformations are shown with RMSD values.

Furthermore, the FEL analysis was performed using the first two PCs (PC1 and PC2) to assess the low-energy structures, shown in (Fig 6) where the deep blue color indicates the global minima. FEL analysis in protein MD simulations is a valuable technique for studying the structural effects of mutations. It provides a comprehensive understanding of the energetic landscape, conformational changes, and functional implications of mutations and offers valuable insights for the design of targeted therapies [49,50]. FEL analysis revealed the presence of three global minima in the WT structure of the 3C protease. The mutants exhibited the occurrence of large transitions during the MD simulations compared to the WT. In particular, the C142L mutation underwent large conformational changes with three global minima distributed over more than three clusters. Moreover, we superimposed the respective global minima structures from all three systems (WT, C142S, and C142L). Interestingly, a clear structural change at the active site β-ribbon structure was observed for the mutants. Again, the superimposition of representative structures from the mutants with the WT revealed an RMSD value of 1.108 Å (168 atoms) and 1.048 Å (174 atoms) against C142S and C142L respectively. All the above analysis indicates that the C142S and C142L mutation may alter the structural conformation particularly the conserved β-ribbon region of the 3C protease. Additionally, it was evident that the cysteine-to-leucine mutation changed the structural organization of the β-ribbon region, which may have an impact on the substrate binding process during proteolysis, whereas the cysteine-to-serine substitution at position 142 may prevent the substrate binding process and inhibit enzymatic activity.

The distinct collective motions observed in the mutants, particularly the increased fluctuation and altered conformation of the β-ribbon region, highlight the significant structural implications of these mutations. The bending of the β-ribbon towards the catalytic pocket in the C142S mutant suggests potential steric hindrance at the substrate binding site, which may inhibit enzymatic activity. On the contrary, the bending of the β-ribbon away from the catalytic pocket in the C142L mutant implies disrupted substrate interactions, likely contributing to reduced enzymatic efficiency. The observed conformational changes, coupled with distinct energy minima distributions, emphasize the role of these mutations in altering the functional dynamics of the protease and provide a basis for understanding their potential effects on substrate binding and proteolysis.

### 3.4. Cross-correlation analysis

**Fig 7:**
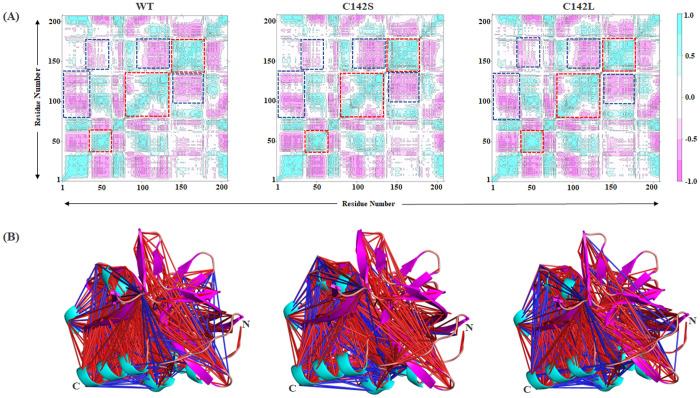
Cross-correlation analysis. (A) Dynamic cross-correlation map (DCCM) of WT and mutants. The red and blue dashed frames represent positive and negative correlations respectively. (B) Structural representation of the strong residual cross-correlation in WT, C142S, and C142L. Red (+1.0) and blue (−1.0) lines denote the most correlated and anticorrelated interactions between two residues. The intensity of the line color denotes the degree of association.

The internal dynamics of the WT and the 3C protease mutants were examined through cross-correlation analysis using the Bio3D package. A dynamic cross-correlation map (DCCM) between wild-type and mutant proteins can provide structural information on how particular mutations affect the correlated motions within individual residue pairs of a protein [51]. Hence, a DCCM map was created using the Cα atoms from the concatenated trajectories (last 100 ns from each replicate; total 300 ns) as shown in Fig 7A. Cyan depicts positively correlated motions (+1.0), while pink depicts negatively correlated motions (−1.0). The color intensity depicts the degree of positive/negative correlations. As shown in Fig 7B, all systems exhibited large correlated and small anti-correlated motions. In particular, the β-ribbon region in WT exhibited only a positive correlation, suggesting that the residues present in this region moved in the same direction only. The C142L mutant system showed less correlated motion and more anti-correlated motion than the WT and C142S systems. Furthermore, the residual motion pattern in the catalytic site was studied by capturing local correlations, and the results suggested that catalytic residues such as H46 and D84 exhibited only positive correlation motion in all systems; C163 exhibited less anti-correlated motion in WT and C142L. In contrast, DCCM analysis revealed that the positive correlation between amino acid residues was much stronger in all systems, whereas the WT and C142L systems showed a similar pattern of anti-correlated motions. These findings imply that the C142L mutation destabilizes the β-ribbon’s synchronized movements, potentially affecting substrate interactions and enzymatic activity, while C142S maintains a motion pattern closer to the WT, with less anti-correlated motion observed in the catalytic residues. This emphasizes the differential impact of the mutations on the protease’s functional dynamics.

### 3.5. Betweenness centrality analysis

**Fig 8:**
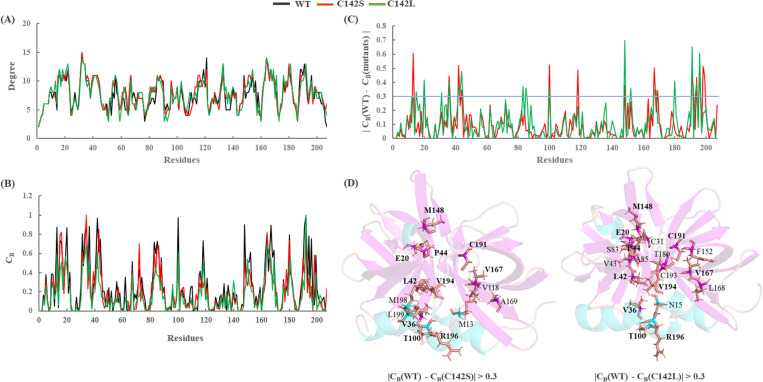
Residue network centrality. (A) Degree centrality (CD); (B) Betweenness centrality (C_B_) are shown for WT, C142S, and C142L; (C) C_B_ difference between WT and mutants; (D) Residues having C_B_ > 0.3 are shown in the stick model and common residues are labeled in bold type. Black, red, and green represent WT, C142S, and C142L models of 3Cpro.

We considered the representative structure of WT and mutants of 3Cpro complexes from the simulation and used them to perform a residue network analysis using NAPS server with a cut-off distance of 0.7 nm (S4A Fig). RIN analysis is a valuable technique for understanding the structural and dynamic properties of proteins and is especially pertinent when comparing wild-type and mutant protein structures. Specific mutations in a protein can affect interactions and connectivity between residues [52–54]. Residue network analysis helps in pinpointing the specific residues or regions that are most affected by mutations. In addition, it provides insight into allosteric communication, which is the transmission of information between distal sites in a protein. Changes in network connectivity between the WT and mutant structures can indicate altered allosteric pathways, helping us to understand the impact of mutations on protein dynamics. The betweenness centrality (C_B_) value was computed using a residual network. This can help to identify residues that play a crucial role in the structural stability or function of a protein. The high C_B_ value of a node may have a significant impact on the structural-functional relationship of the complex. Subsequently, we calculated the degree of centrality (C_D_) and C_B_ differences between the WT and mutant complexes represented in Fig 8A and 8B.

The condition |C_B_(WT)−C_B_(mutant)| > 0.3 was used to map the respective amino acids on the protein structures to understand the residual interaction variations between WT and mutants. From this analysis, we found that the residues in the interior surface region of the β-barrels of 3Cpro exhibited larger C_B_ values than other regions, suggesting this region might be necessary for signal transmission (S4B Fig). We also observed similar patterns of essential residues in both mutants. More than 50% of the residues were found to be common among the mutants (Fig 8C and 8D), and the non-common residues suggest that intra-residual signaling differs in the mutant complexes as compared to the WT 3Cpro, thereby impacting the function of the protein. In particular, the central region of 3Cpro, accommodating the active site and catalytic triad, may play an important role in the conformational shift required for substrate binding. However, additional studies are required to validate the functions of these residues.

**Fig 9:**
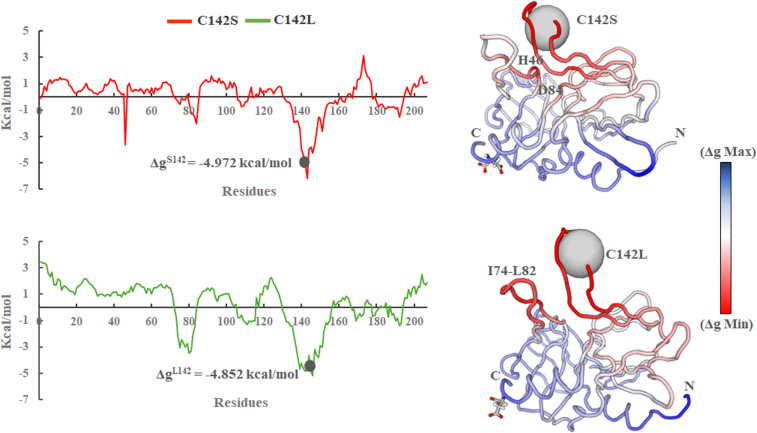
Free energy of each residue in response to both mutations. The red and blue color in the protein’s structure represent the most stabilized and destabilized regions respectively. Residues H46 and D84 in the C142S mutant and residues I74 to L82 in the C142L mutant model revealed a strong stabilized region.

Furthermore, the free energy (Δg) of each residue in response to a specified mutation was computed using the representative structure and AlloSigMA server [55]. It is used to calculate the allosteric free energy experienced by a single residue due to mutations. Allosteric free energy was used to assess the allosteric communication linked to a mutation. Specifically, a positive sign of the allosteric free energy indicates destabilized residues, whereas a negative sign indicates stabilized residues, both of which lead to local stabilization [56,57]. The results revealed that residues in the β-ribbon region are stabilizing with negative free energy (−Δg) for both mutations. The C142S and C142L mutations revealed free energy values of −4.972 and −4.852 Kcal/mol, respectively. Although catalytic residues such as H46 and D84 were found to be less stabilized in the C142L mutant, the residues present near D84 (residues 74–82) revealed a strongly stabilized beta-sheet compared to C142S (Fig 9). In contrast, compared to C142L, the C142S mutant exhibited stable catalytic and active site regions indicating that side chains of β-ribbon residues may be involved in intra-residual activity to form a locked active site conformation for substrate binding. Hence, it is possible that a single mutation at position 142 may potentially control the protein’s function through an allosteric mechanism.

## 4. Conclusion

The 3C protease (3Cpro) is one of the most important genes in the viral genome of FMDV and is responsible for 10 of 13 cleavages by targeting specific sequences in its polyprotein structure. The crucial role of 3Cpro in proteolytic activity and viral replication makes it a suitable drug target for the development of various therapeutics against FMD. Previous studies reported that, despite of having a degree of flexibility, the conserved β-ribbon structure containing the active site residues C142 contributes significantly to substrate specificity. Mutagenesis of C142 at the apical-tip of the β-ribbon structure significantly affects catalytic activity. However, the underlying allosteric molecular mechanisms of these mutations remain unclear, limiting the development of novel 3Cpro inhibitors and how these mutations in the active site affect enzyme activity. The MD simulation analysis with three different replicates presented in the current study deciphered the conformational alterations that occur upon active-site mutations of 3Cpro. The all-atom MD simulation results suggested greater structural stability in the WT and C142L than in C142S. Although there were no significant changes observed in the number of intra-hydrogen bonds and Rg values of WT and mutants, the global dynamics analysis revealed large and opposite structural deviation of the conserved β-ribbon in both the mutants, compared to the WT complex. In the case of the C142L mutant system, the β-ribbon structure is observed to bend away from the catalytic pocket while in the case of the C142S mutant, it bends towards the catalytic pocket to form an open and closed conformation respectively. Residual cross-correlation analysis revealed a strong positive correlation between the amino acid residues of all three systems. In particular, WT and C142L mutants exhibited similar patterns of anti-correlated motions between residues. Moreover, the difference in the free energies (Δg) values of each residue and betweenness centrality from RINs indicates that a single mutation in the active site of 3Cpro affects the dynamics of the protein which may result in allosteric regulation of its biological activity. Additionally, it was suggested that significant residues might be accountable for the functional variation in the mutants relative to the WT. From these findings, we believe that active-site mutations, particularly C142S, induce specific structural and conformational changes that inhibit protein activity. By combining the results obtained from this study with targeted experimental mutagenesis, it will be plausible to provide a detailed understanding of the allosteric mechanisms induced by these mutations which will be helpful in the development of novel therapeutics against FMD.

## Supporting information

S1 Fig Validation of AlphaFold2 modeled structure of FMDV 3C protease (WT, C142S, and C142L) using Ramachandran plot. (TIF)

S2 Fig Evolutionary conservation profile of 3Cpro. (TIF)

S3 Fig Collective mode analysis. (A) RMSIP of the first 10 PCs showing similarities and dissimilarities between WT and mutants. (B) The 2D projection of PC1 vs. PC2, PC2 vs. PC3, and PC1 vs. PC3. Black, red, and green colors represent the WT, C142S, and C142L systems of 3Cpro.
(TIF)

S4 Fig Network topology. (A) Residue interaction networks were constructed for all the systems using a cut-off of 0.7nm. The mutations were highlighted in red color. (B) The table represents the residues for which the CB > 0.3 between WT and mutants. Bold types represent the common residues in both mutants.
(TIF)

S1 Table DSSP analysis of WT and mutants representing the overall percentage changes in their secondary structure. (DOCX)
