# Supplementary figures and images for: Elucidating the structural dynamics induced by active site mutations in 3C protease of foot-and-mouth disease virus
Source: PLoS One. 2025 Apr 21;20(4):e0321079. doi: 10.1371/journal.pone.0321079 (PMC12011219; doi:10.1371/journal.pone.0321079)

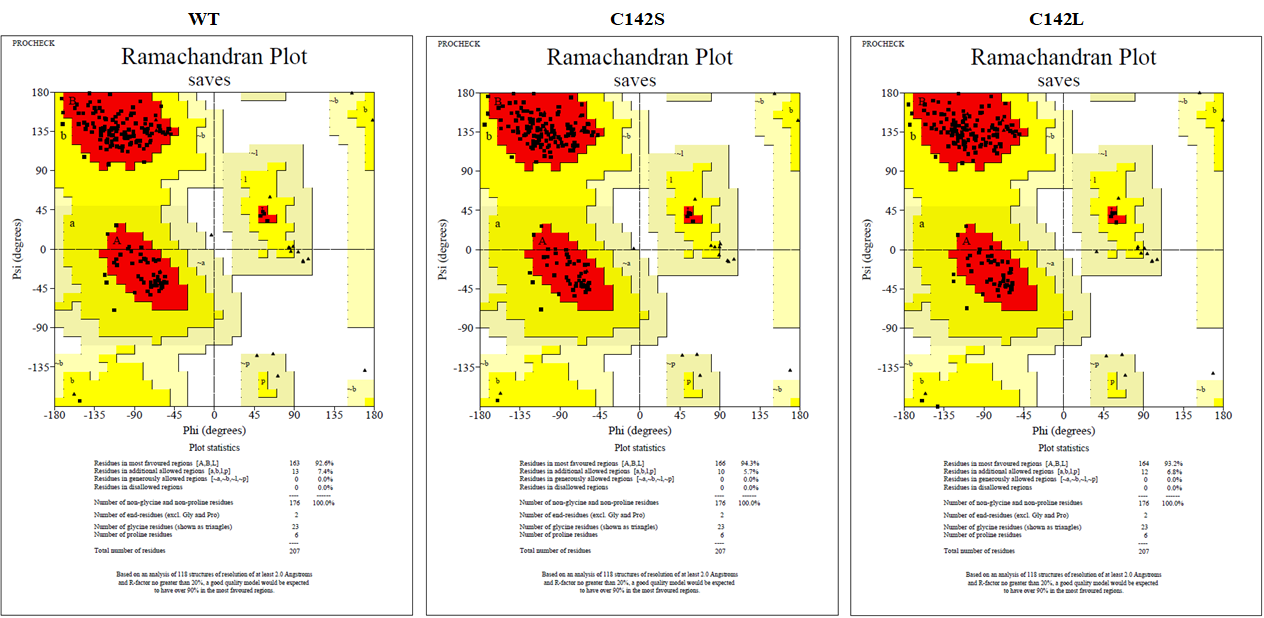

Supplement: S1 Fig — (TIF) [file pone.0321079.s001.tif]

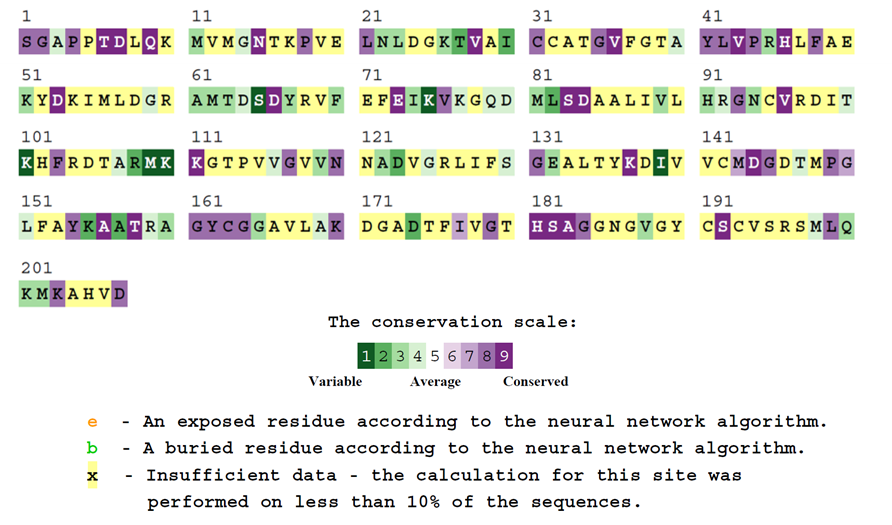

Supplement: S2 Fig — (TIF) [file pone.0321079.s002.tif]

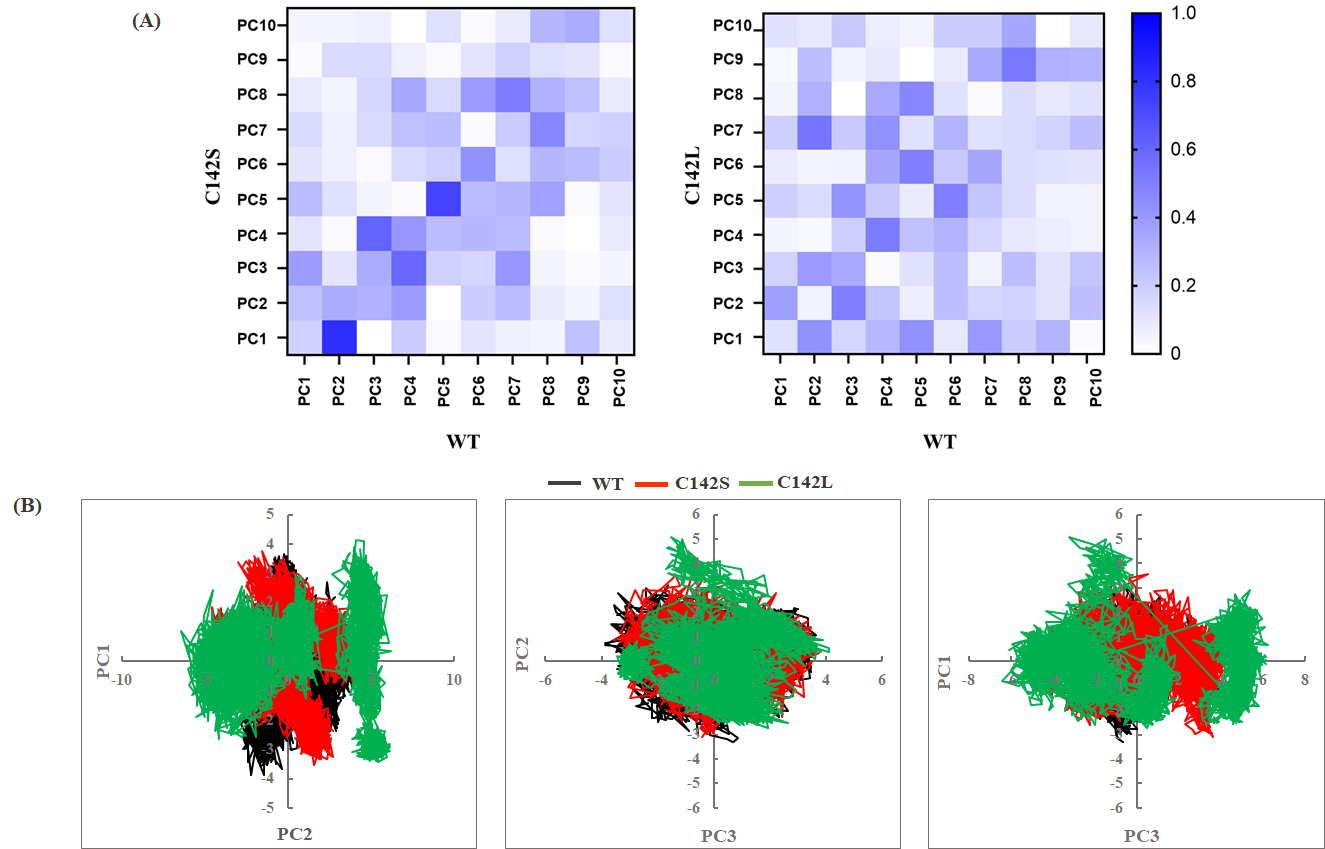

Supplement: S3 Fig — (A) RMSIP of the first 10 PCs showing similarities and dissimilarities between WT and mutants. (B) The 2D projection of PC1 vs. PC2, PC2 vs. PC3, and PC1 vs. PC3. Black, red, and green colors represent the WT, C142S, and C142L systems of 3Cpro. (TIF) [file pone.0321079.s003.tif]

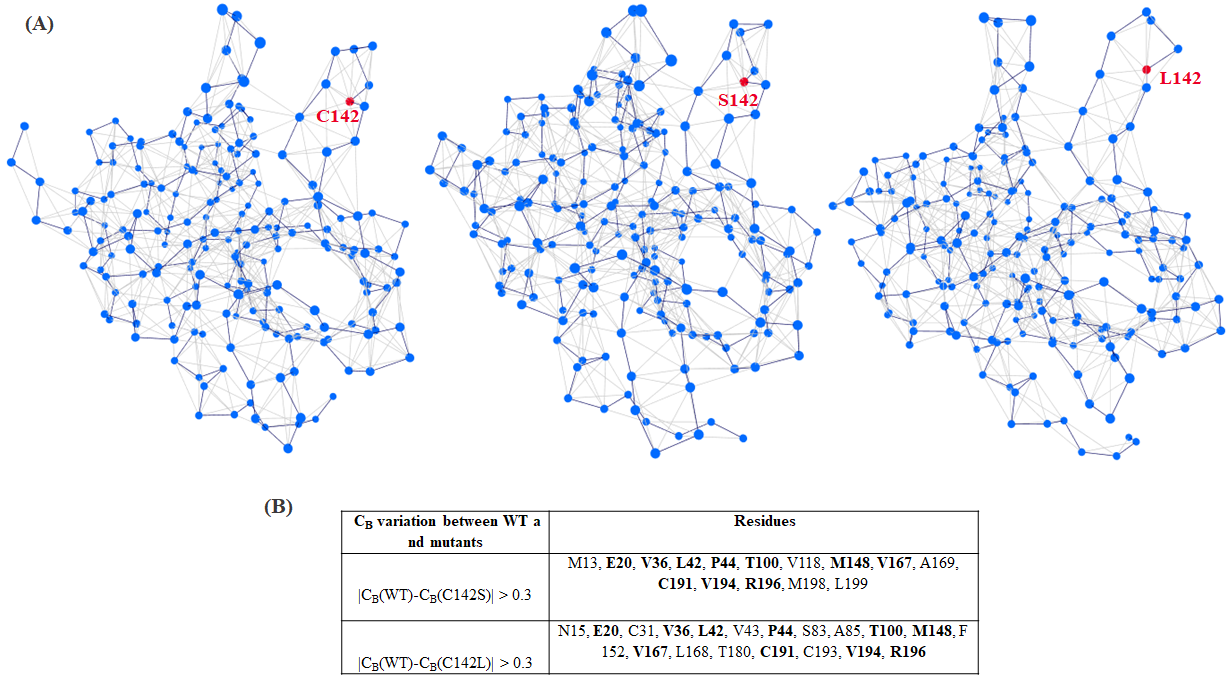

Supplement: S4 Fig — (A) Residue interaction networks were constructed for all the systems using a cut-off of 0.7nm. The mutations were highlighted in red color. (B) The table represents the residues for which the CB > 0.3 between WT and mutants. Bold types represent the common residues in both mutants. (TIF) [file pone.0321079.s004.tif]
