# Supplementary material for: Elucidating the structural dynamics induced by active site mutations in 3C protease of foot-and-mouth disease virus
Source: PLoS One. 2025 Apr 21;20(4):e0321079. doi: 10.1371/journal.pone.0321079 (PMC12011219; doi:10.1371/journal.pone.0321079)
Supplement: S1 Table — (DOCX) [file pone.0321079.s005.docx]

S1 Table. DSSP analysis of WT and mutants representing the overall percentage changes in their secondary structure.

| **System** | **Coil** | **B-Sheet** | **B-Bridge** | **Bend** | **Turn** | **A-Helix** | **3-Helix** | **5-Helix** |
| --- | --- | --- | --- | --- | --- | --- | --- | --- |
| **WT** | 17.69675 | 50.76402 | 0.771661 | 6.014131 | 12.47438 | 8.108868472 | 4.170183 |  |
| **C142S** | 17.90816 | 51.04054 | 0.942069 | 6.512495 | 12.01219 | 8.797736 | 2.786821 |  |
| **C142L** | 17.79643 | 51.17558 | 0.93805 | 6.255275 | 12.07167 | 7.850041 | 3.904925 | 0.008038 |
